# Late Administration of Surfactant May Increase the Risk of Patent Ductus Arteriosus

**DOI:** 10.3389/fped.2020.00130

**Published:** 2020-03-31

**Authors:** Fuat Emre Canpolat, Gülsüm Kadıoğlu Şimşek, James Webbe, Mehmet Büyüktiryaki, Nazmiye Bengü Karaçağlar, Sarkhan Elbayiyev, H. Gözde Kanmaz Kutman

**Affiliations:** ^1^NICU, Department of Neonatology, Ankara City Hospital, University of Health Sciences, Ankara, Turkey; ^2^Neonatal Medicine, Imperial College London, London, United Kingdom

**Keywords:** surfactant, early rescue, patent ductus arteriosus, respiratory distress syndrome, preterm infant

## Abstract

**Introduction:** Early rescue surfactant is the most effective way of administering surfactant but many infants still receive surfactant later. Our aim was to explore the association between timing of surfactant administration and the development of patent ductus arteriosus and other neonatal morbidities.

**Materials and method:** This retrospective study analyzed 819 preterm infants under 30 weeks of gestational age and under 1,500 g.

**Results:** Five hundred and ninety three infants received surfactant during the study period, of these 365 received it within 2 h of life (early group) and 228 received it after two h (late group). Patent ductus arteriosus was detected in 175 (48%) of the early group and 168 (74%) of the late group, *p* = 0.001. Multinominal logistic regression analysis demonstrated that receiving surfactant after 2 h of life has a OR 3.5 (2.2–5.64 95 % CI) and a *p*-value of 0.001 for developing patent ductus arteriosus.

**Conclusion:** In this study population we observed that late surfactant administration is associated with increased risk of patent ductus arteriosus.

## Introduction

Respiratory distress syndrome (RDS) is the most common respiratory disease among preterm infants. Surfactant replacement therapy is an effective treatment for respiratory distress syndrome (1). Surfactant therapy has been shown to reduce disease severity and air leaks leading to improved survival (1). Although management has evolved gradually over the years early rescue surfactant administration is superior to late treatment (2). Previous trials showed that surfactant given earlier in the course of disease works better than later in terms of reducing air leaks (2) and avoiding mechanical ventilation if the intubate-surfactant-extubate (INSURE) technique is used (3). European Consensus Guideline 2019 recommended that early rescue surfactant should be standard (1) and there are occasions when surfactant should be given in the delivery suite, such as when intubation is needed for stabilization.

In some previous reports, early administration of surfactant is associated with shorter duration of ventilation, longer duration of continuous positive airway pressure and longer hospital stay but had little or no impact on bronchopulmonary dysplasia and/or mortality (4). There are limited and controversial data about the effect of surfactant timing on patent ductus arteriosus (5, 6). One recent trial with a large number of patients concluded that neonates in the late surfactant group who received more than one dose of surfactant were at higher risk of bronchopulmonary dysplasia, retinopathy of prematurity and patent ductus arteriosus requiring ligation (7).

Therefore, our aim was to investigate the relationship between timing of surfactant and development of patent ductus arteriosus. A secondary aim was to compare other neonatal morbidities among these groups.

## Method

We retrospectively analyzed 819 cases of infants hospitalized in our neonatal intensive care unit during the study period (the years 2013–2018): 593 of these babies received surfactant. The infants were born at <30 weeks of gestation and had birth weight ≤1,500 g. We divided the patients into two groups based on timing of surfactant therapy: the early surfactant group (365 infants) receiving surfactant within 2 h of life and the late group (228 infants) receiving it after 2 h.

In this retrospective cohort, preterm infants with major congenital anomalies, infants with futile or palliative care, and missing information about surfactant administration time were excluded from the study.

Although the FiO_2_ requirement >30% in infants with respiratory distress syndrome findings is considered to be an important predictor of the severity of respiratory distress syndrome and non-invasive ventilation failure, considering the high surfactant treatment rates in Turkey, we used administering surfactant in infants who require ≥40% FiO_2_ during the study period (2013–2018) as our Turkish Guideline recommended (8). All very low birth weight preterm infants received continuous positive airway pressure (CPAP) as initial respiratory support in the delivery room.

### Definitions of Outcomes

Echocardiographic criteria for a diagnosis of patent ductus arteriosus (PDA) were duct size >1.5 mm, a left atrium-to aortic root (LA:Ao) >1.5, left-to-right shunting of blood, end-diastolic reversal of blood flow in the aorta or poor cardiac function in addition to signs of PDA. Two-dimensional color Doppler echocardiography was performed using a GE Vivid 7 Pro, 10S transducer (GE Healthcare, Salt Lake City, Utah). Patent ductus arteriosus was defined as the requirement for medical treatment with evidence of echocardiographic findings at least once at any time during the NICU (Neonatal Intensive Care Unit) admission.

Cranial ultrasound imaging results were based on the worst finding at any given time for this patient before discharge: intraventricular hemorrhage grade III/IV was defined as intraventricular hemorrhage with ventricular enlargement as Papile et al. reported (9). Early onset sepsis was defined as isolation of bacterial, fungal, or viral organism from blood or cerebrospinal fluid in a symptomatic infant within 3 day of life and if more than 3 days defined as late onset sepsis. Bronchopulmonary dysplasia was defined as receiving supplemental oxygen at 36 weeks postmenstrual age (10). Retinopathy of Prematurity was defined according to the International Classification of Retinopathy of Prematurity (ICROP) (11). Clinical chorioamnionitis was defined as inflammation of the chorion and amnion evidenced by presence of either maternal fever ≥38.4°C within 24 h before birth (irrespective of epidural analgesia), uterine tenderness, maternal leukocytosis of >15,000/mm^3^, or explicitly mentioned obstetric concern at delivery. Information about histologic confirmation was not available. Days of oxygen, continuous positive airway pressure, or mechanical ventilation were counted during the primary NICU stay before discharge. Treatment of respiratory distress syndrome in our center was according to our local guidelines. Our center generally attempts to minimize invasive respiratory support with initiation of continuous positive airway pressure for all infants at risk of respiratory distress syndrome as soon as possible after birth in the delivery room, with the exception of extremely low gestational ages such as those who are <26 weeks. Continuous positive airway pressure support is the first line of respiratory support with the option of nasal intermittent mandatory ventilation or nasal intermittent positive pressure ventilation in case of increasing episodes of apnea and increased oxygen requirements. Intubation and surfactant administration follows the early symptomatic approach supported by large trials, generally with a cut-off of around 0.4 FiO_2_ with concomitant radiological changes, increased work of breathing and evidence of respiratory acidosis with pH < 7.25 and pCO_2_ >60 mmHg, followed by conventional mechanical ventilation with high frequency modes used generally as rescue therapy. We generally use less invasive surfactant administration procedure to give surfactant to spontaneously breathing preterms (12).

### Statistical Analysis

Baseline demographics and the neonatal outcomes were compared among two surfactant timing groups: infants with early and late surfactant, using Chi-square test for categorical variables and Student *t*-test for continuous variables. To further examine the effect of late surfactant use, multiple logistic regression models were applied to compare the primary and secondary outcomes between the surfactant timing groups. The odds ratio (95% confidence interval) of outcomes were determined based on the final multiple logistic regression models derived by backward variable selection procedure with inclusion criterion of 0.05. The factors for the full model included gender, cesarean section, antenatal steroid use as factors, gestational age, and birth weight as covariates. The data management and all statistical analyses were performed using SPSS for Windows version 22, IBM (USA). A two-sided significant level of 0.05 was used.

## Results

**Figure 1:**
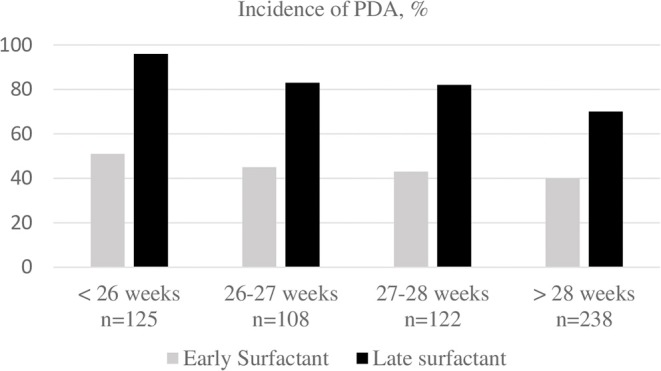
Patent ductus arteriosus incidence among gestational ages compared by early vs. late surfactant timing. PDA, patent ductus arteriosus.

After exclusion, 819 preterm infants were analyzed in this retrospective study. Five hundred and ninety three infants received surfactant during the years 2013–2018, of these 365 received it within 2 h of life (early rescue group, first group) and 228 received it after two h (late group, second group). Mean gestational age was 27 ± 1.7 and 27.3 ± 1.5 (*p* = 0.1) weeks and mean birth weight was 945 ± 248 vs. 974 ± 232 g (*p* = 0.25), respectively. In the first group 186 (51%) and 111 (49%) in second group were boys. Antenatal steroid ratio was 68% in early and 73% in late group. Other obstetric risks were similar and shown in Table 1. Patent ductus arteriosus was detected in 175 (48%) of the early group and 168 (74%) of the late group, *p* = 0.001. All other early neonatal outcomes were compared in Table 2. Multinominal logistic regression analysis was made with antenatal steroids (even no difference between groups) and receiving surfactant (any time) as risk factors and gestational age and birth weight as covariates. This analysis found that receiving surfactant after 2 h of life has a OR 3.5 (2.2–5.64 95 % CI) and a *p*-value of 0.001 for developing patent ductus arteriosus. We grouped and analyzed patients according to gestational age and this is demonstrated in Figure 1.

**Table 1:** Basic clinical characteristics of study groups.

	**Early surfactant *n* = 365**	**Late surfactant *n* = 228**	***p***
Gestational age, weeks ± SD	27 ± 1.7	27.3 ± 1.5	0.1
Birth weight, g ± SD	945 ± 248	974 ± 232	0.25
Male gender, *n* (%)	186 (51)	111 (49)	0.64
Antenatal steroids, *n* (%)	248 (68)	166 (73)	0.3
Chorioamnionitis, *n* (%)	43 (12)	20 (9)	0.251
Preeclampsia, *n* (%)	69 (19)	38 (17)	0.77
Gestational diabetes, *n* (%)	14 (4)	7 (3)	1.0
Cesarean section, *n* (%)	292 (80)	175 (77)	0.43
Multiple gestation, *n* (%)	80 (22)	48 (21)	0.494
Rupture of membranes, *n* (%)	70 (19)	29 (13)	0.182

**Table 2:** Comparison of clinical outcomes of study population.

	**Early surfactant *n* = 365**	**Late surfactant *n* = 228**	***p***
Apgar score	5 (2–8)	5 (1–8)	0.827^*^
Last day of life receiving oxygen	42 ± 24	40 ± 21	0.597
Respiratory support at 28th day			
Supplemental oxygen	186 (51)	120 (53)	0.639
Non-invasive ventilation	76 (21)	45 (20)	
nIMV	55 (15)	38 (16)	
Mechanical ventilation	54 (15)	32 (14)	
Last day of life receiving respiratory support, days ± SD	21 ± 18	19 ± 15	0.373
Duration of CPAP, days ± SD	5.7 ± 6	7.6 ± 7	0.011
Duration of nIMV, days ± SD	3.1 ± 5	3.8 ± 5	0.236
Duration of MV, days ± SD	6.2 ± 10	7.7 ± 15	0.251
Air leaks, *n* (%)	10 (2.7)	8 (3.5)	0.282
PDA, *n* (%)	175 (48)	168 (74)	0.001
Surfactant more than 2 doses	95 (26)	68 (30)	0.313
Early neonatal sepsis, *n* (%)	80 (22)	57 (25)	0.386
Late onset sepsis, *n* (%)	131 (36)	75 (33)	0.456
BPD, moderate and severe, *n* (%)	58 (16)	38 (17)	0.8
IVH Grade III and IV, *n* (%)	58 (16)	27 (12)	0.17
ROP, *n* (%)	36 (10)	32 (14)	0.12
Day of discharge, days ± SD	69 ± 21	70 ± 22	0.68
Mortality, *n* (%)	36 (10)	29 (11)	0.182

*CPAP, continuous positive airway pressure; nIMV, nasal intermittent mandatory ventilation; MV, mechanical ventilation; PDA, patent ductus arteriosus; BPD, bronchopulmonary dysplasia; IVH, intraventricular hemorrhage; ROP, retinopathy of prematurity; SD, standard deviation*;

* *non-parametric test*.

## Discussion

According to the results of this study, administering surfactant after the first 2 h of life is associated with higher frequency of patent ductus arteriosus. Although similar studies have been published previously (6, 7), this study is the first in the literature to draw attention to this subject. In addition, the duration of continuous positive airway pressure was slightly but statistically longer in the group with late surfactant administration compared to the group with early administration.

Surfactant therapy is now accepted as the standard treatment method for respiratory distress syndrome and reduces mortality (1, 8). Recommendations for surfactant therapy include preferring natural surfactant preparations, applying it as targeted early rescue therapy, and administering by non-invasive methods if the patient is breathing spontaneously (1, 8).

However, the principles of surfactant administration may vary from country to country or between different guidelines (1, 6, 8, 13, 14). The available evidence suggests that if the patient needs surfactant administering it as early as possible is most beneficial (2, 15). There is also evidence on non-invasive ventilation in the first 72 h of life reduces the need for invasive ventilation and surfactant requirement (16), these all evidences are a part of need for surfactant. The advantages of early surfactant administration over late administration can be summarized as lower incidence of air leaks, less need for mechanical ventilation and a decreased risk of neonatal mortality and bronchopulmonary dysplasia (1, 2). In recent years, decisions regarding whether or not to administer surfactant therapy have been based primarily on an oxygen requirement threshold according to international guidelines (1). This threshold, which is 40% according to old guidelines, has also been applied in Turkey and in the present study (8). In more recent guidelines, this figure has been reduced to 30%, which suggests that surfactant will now be given much earlier and can be interpreted as promoting early surfactant administration (1).

Although early surfactant administration is recommended based on the findings of numerous trials and other studies, it is not always possible in clinical practice. Some patients may have very good clinical condition, normal or near-normal x-rays, low oxygen requirement, and very little need of respiratory support in the first hours. For reasons that are not well-understood, at postnatal age of 6–12 h or later these infants show increased oxygen requirement that cannot be met by pressure support ventilation, and the need for surfactant can arise even though it was not needed before.

This may be attributed to several factors, including the conditions of postnatal care, effectiveness of continuous positive airway pressure, the interface, and depletion and inadequacy of the infant's endogenous surfactant. If continuous positive airway pressure is not effective enough, the infant loses alveolar patency in a matter of hours and has higher tendency toward hypoxia, resulting in lung parenchyma that requires surfactant. With the administration of surfactant in addition to the effects of hypoxia on the lungs, there is a sudden reduction in pulmonary pressure and the ductus arteriosus remains patent. Surfactant administered at a later time causes sudden hemodynamic changes in a lung that has gradually deteriorated and entered this vicious cycle within hours.

This study has certain limitations. One of these is its retrospective nature. Furthermore, the fact that it was conducted at a single center based on a single guideline can also be considered a limitation. Nevertheless, our patient sample is sufficient, and to the best of our knowledge, there is no other study in the literature examining the impact of late surfactant administration on patent ductus arteriosus in a patient group of this size.

In conclusion, our findings support the well-established practice of early surfactant administration from a different angle. Early surfactant administration leads to a lower incidence of patent ductus arteriosus and reduced the need for respiratory support (continuous positive airway pressure duration) in neonates. We did not observe any differences between the groups in terms of bronchopulmonary dysplasia or other morbidities, but prospective studies based on new guidelines may provide stronger evidence as to whether such differences exist.

## Data Availability Statement

The datasets generated for this study are available on request to the corresponding author.

## Ethics Statement

The studies involving human participants were reviewed and approved by Zekai Tahir Burak Hospital Ethical Committee. Written informed consent to participate in this study was provided by the participants' legal guardian/next of kin.

## Author Contributions

FC, GK, and MB collected data. NK and SE patient management, data management, and entering data. GK and FC crtitcal review. HK and FC made statistical analysis. JW made review.

### Conflict of Interest

The authors declare that the research was conducted in the absence of any commercial or financial relationships that could be construed as a potential conflict of interest.
